# Conversion of Roux-en-Y gastric bypass to single anastomosis duodenal ileal bypass with sleeve gastrectomy with gastrogastric jejunal bridge

**DOI:** 10.1016/j.mex.2022.101971

**Published:** 2022-12-16

**Authors:** Victor Ramos Mussa Dib, Carlos Augusto Scussel Madalosso, Gabriela Trentin Scortegagna, Rui Ribeiro

**Affiliations:** a Victor Dib Institute, 1444 Álvaro Botelho Maia Ave, 69020-210, Manaus, AM, Brazil; b Gastrobese Clinic, 1953 Uruguai St., 8th floor, 99010-111, Passo Fundo, RS, Brazil; c Hospital Lusíadas Amadora, 8 Hospitais Civis de Lisboa Ave, 2724-002, Amadora, Lisbon, Portugal

**Keywords:** Gastric Bypass, Roux-en-Y Anastomosis, Biliopancreatic Diversion, Obesity Recidivism, Conversion of RYGB to SADIs with Gastrogastric Jejunal Bridge

## Abstract

Surgical conversion of Roux-en-Y gastric bypass (RYGB) to one anastomosis duodenal switch with sleeve gastrectomy (SADI-S), can be effective, when there is obesity recidivism, but surgically challenging. This case report video aims to detail the technical modifications that simplifies this conversion, in one stage. This video article demonstrates the conversion of RYGB to SADI-S using a jejunal bridge to facilitating the gastro-gastric reconnection. Surgical conversion was done laparoscopically, firstly removing the fundus, gastric body and the proximal part of the antrum. The gastrojejunal (GJ) anastomosis from the previous RYGB was preserved and the jejunal alimentary limb that follows was transected, 8cm distal to the GJ anastomosis, and anastomosed, at this level, with the antrum. The remaining alimentary limb was removed, until the jejuno-jejuno anastomosis, from the previous RYGB. The interposition of a segment of jejunal alimentary limb between the gastric bypass pouch and the antrum, has shown to be safe and feasible in RYGB conversion to SADI-S, without complications. Not reconnecting the remnant jejunal alimentary limb to the intestinal transit, but removing it, makes the procedure shorter and safer.

## Graphical abstract

**Figure:**
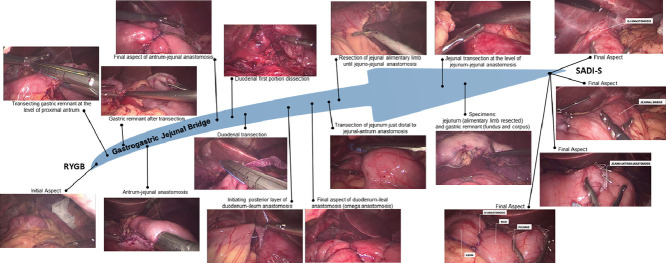

Specifications table

**Table:** 

Subject area:	Medicine and Dentistry
More specific subject area:	Bariatric Surgery
Name of your protocol:	Conversion of RYGB to SADIs with Gastrogastric Jejunal Bridge
Reagents/tools:	NA
Experimental design:	The surgical conversion was performed laparoscopically, preserving the gastrojejunal anastomosis, from RYGB, using the proximal part of the jejunal alimentary limb, which was sectioned 8 cm distally to the gastrojejunal anastomosis and was used as a bridge between the gastric pouch and the antrum. Before that, the resection of the fundus, corpus and part of the antrum was done. This was performed in a single stage procedure.
Trial registration:	NA
Ethics:	An informed consent signed by the patient was obtained to the use of case details and images of laparoscopic surgery and endoscopy by the surgeon.
Value of the Protocol:	• Demonstration of conversion from RYGB to SADI-S by jejunal bridge and resection of RYGB's remaining alimentary segment. • Technical modification that reduces surgical time and makes the surgery simpler, feasible and safer. • Good option for cases in which gastric reservoir of RYGB is ultra-short and/or when adhesions in GJ anastomosis region is very intense.

## Description of protocol

Roux-en-Y gastric bypass (RYGB) for the treatment of morbid obesity is still a very prevalent surgery in the world (1), but with some long-term obesity recurrence rate (about 20 to 30%) (2). Therefore, some of these patients need surgical reinterventions and conversions. However, the technical complexity of these conversions, with multiple resections and anastomosis, must be considered (3). Surgical conversion to techniques with greater metabolic power, such as one anastomosis duodenal switch with sleeve gastrectomy (SADI-S), can be effective in this condition, but represents a technical challenge, especially if the gastric pouch of the primary procedure is short (4). This video article (*link1*) aims to demonstrate in details some technical modifications in the conversion of RYGB to SADI-S, in a patient with obesity recidivism after RYGB, that makes this procedure simpler and safer, feasible in a single stage, by reducing the number of anastomosis and diminishing its risks and complexity. The surgical team, patient, and trocars positions during the procedure, and the clinical aspects of this case report were recently published (5). This patient had chronic cholecystitis, and candy cane and had cholecystectomy and candy cane removal during the procedure (not shown in the video). Here we listed the specific steps to perform the conversion of RYGB to SADI-S with gastrogastric jejunal bridge. Watch the video (*link1*) to visualize the step-by-step described below highlighting the innovative surgical strategy proposal.

## Surgery process steps

**Fig. 1:**
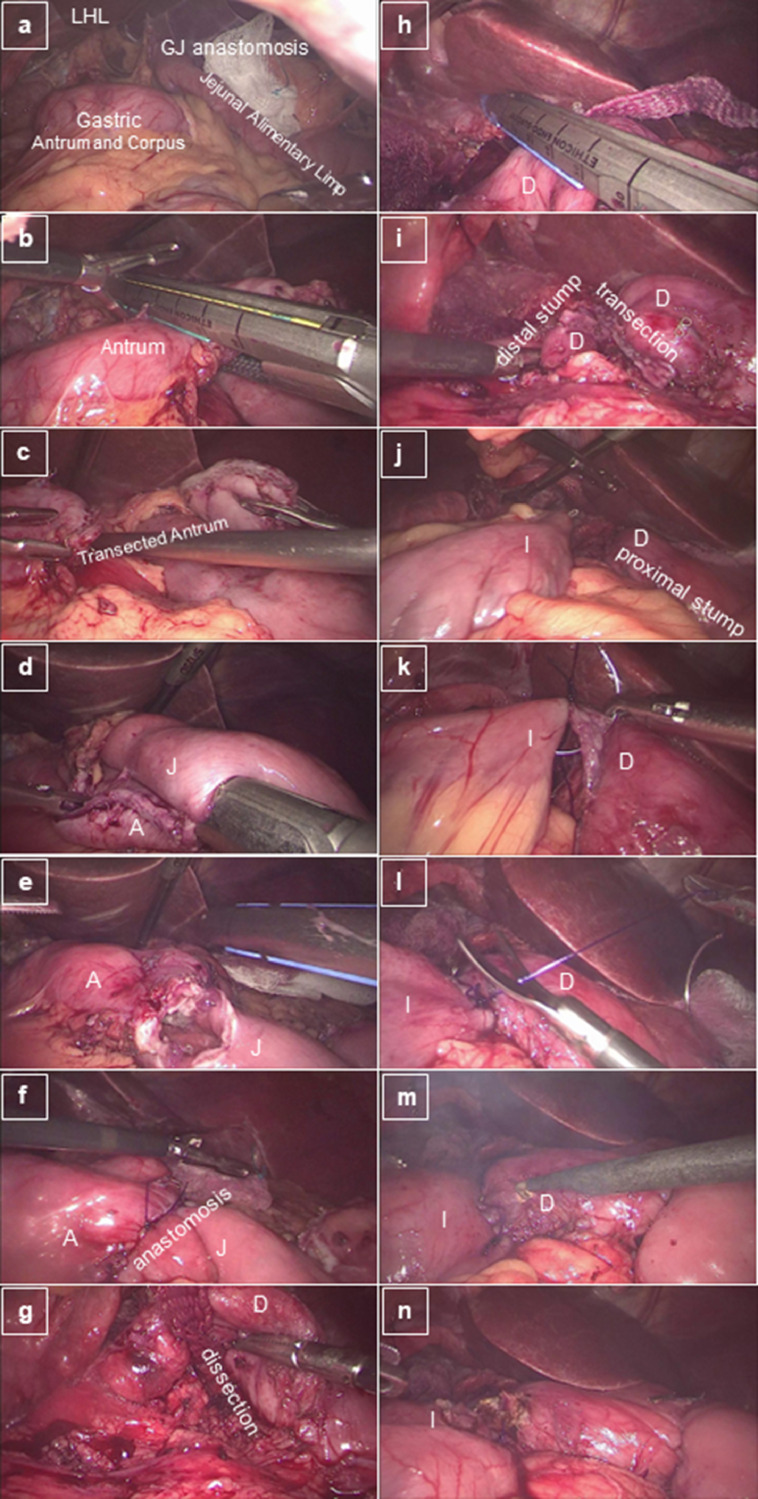
Sequence of the laparoscopic conversion of RYGB to SADIs with gastrogastric jejunal bridge. Part 1 – from identification of the gastroentero anastomosis to opening duodenum and ileum. (a) Initial aspect; (b) Transecting gastric remnant at the level of proximal antrum; (c) Gastric remnant after transection; (d) Antrum-jejunal anastomosis; (e) Aspect after antrum-jenunal stapling; (f) Final aspect of Jejuno Antrum Anastomosis; (g) Duodenal first portion dissection; (h) Duodenal transection; (i) Aspect after duodenal transection; (j) Bringing the ileum to the proximal duodenum stump; (k) Initiating posterior layer of duodenum-ileum anastomosis; (l) Finishing posterior layer of duodenum-ileum anastomosis; (m) Opening duodenum and ileum for the anastomosis; and (n) After opening duodenum and ileum; *LHL* Left Hepatic Lobe; *GJ* Gastro-Jejunal; *A* Antrum; *J* Jejun; *D* Duodenum; *I* Ileum;

**Fig. 2:**
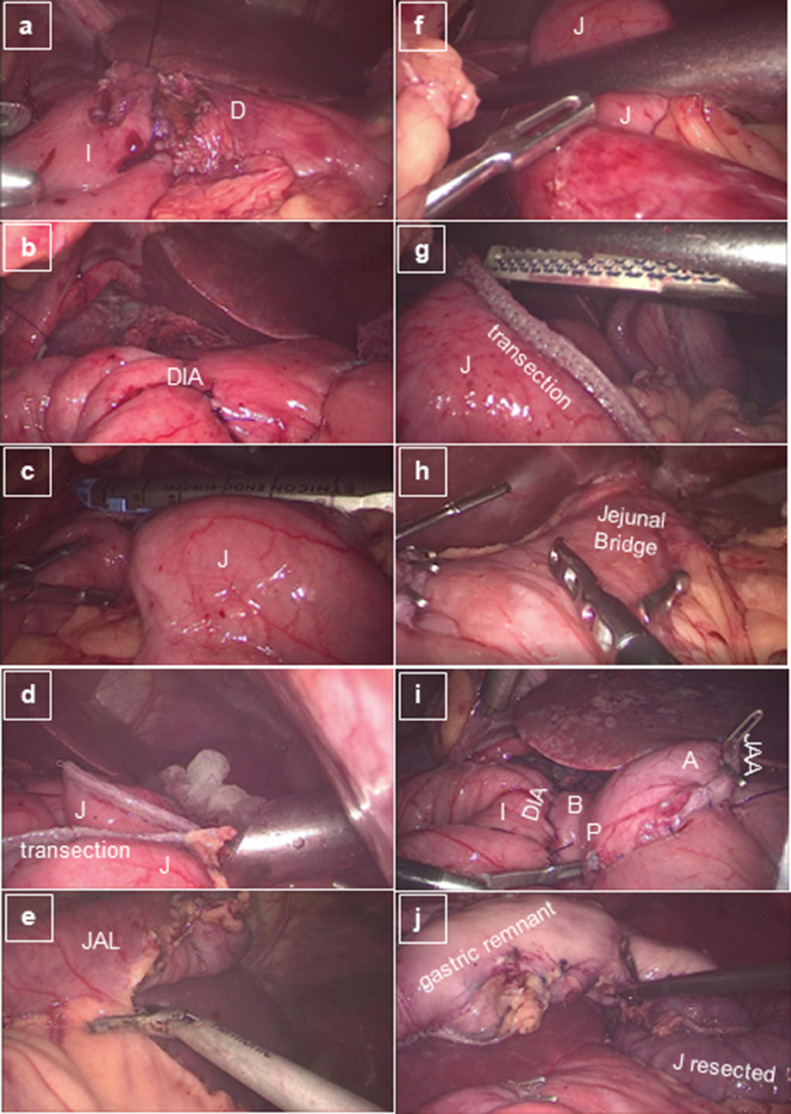
Sequence of the laparoscopic conversion of RYGB to SADIs with gastrogastric jejunal bridge. Part 2 – from duodenum-ileum anastomosis to finial aspect of conversion. (a) Initiating anterior layer of duodenum-ileum anastomosis; (b) Final aspect of duodenum-ileal anastomosis (omega anastomosis); (c) Transection of jejunum just distal to jejunal-antrum anastomosis; (d) Aspect after jejunum transection; (e) Resection of jejunal alimentary limb until jejuno-jejunal anastomosis; (f) Jejunal transection at the level of jejunum-jejunal anastomosis; (g) Aspect after jejunal transection at the level of jejunum jejunal anastomosis; (h) Jejunal Bridge; (i) Aspect of jejunum-antrum anastomosis and duodenum-ileal anastomosis; (j) Specimens: jejunum (alimentary limb resected) and gastric remnant (fundus and corpus). *A* Antrum; *J* Jejun; *D* Duodenum; *I* Ileum; *JAL* Jejunal Alimentary Limb; *JAA* Jejunum-Antrum Anastomosis; *P* Pylorus; *B* Bulb; *DIA* duodenum-ileal anastomosis;

**Fig. 3:**
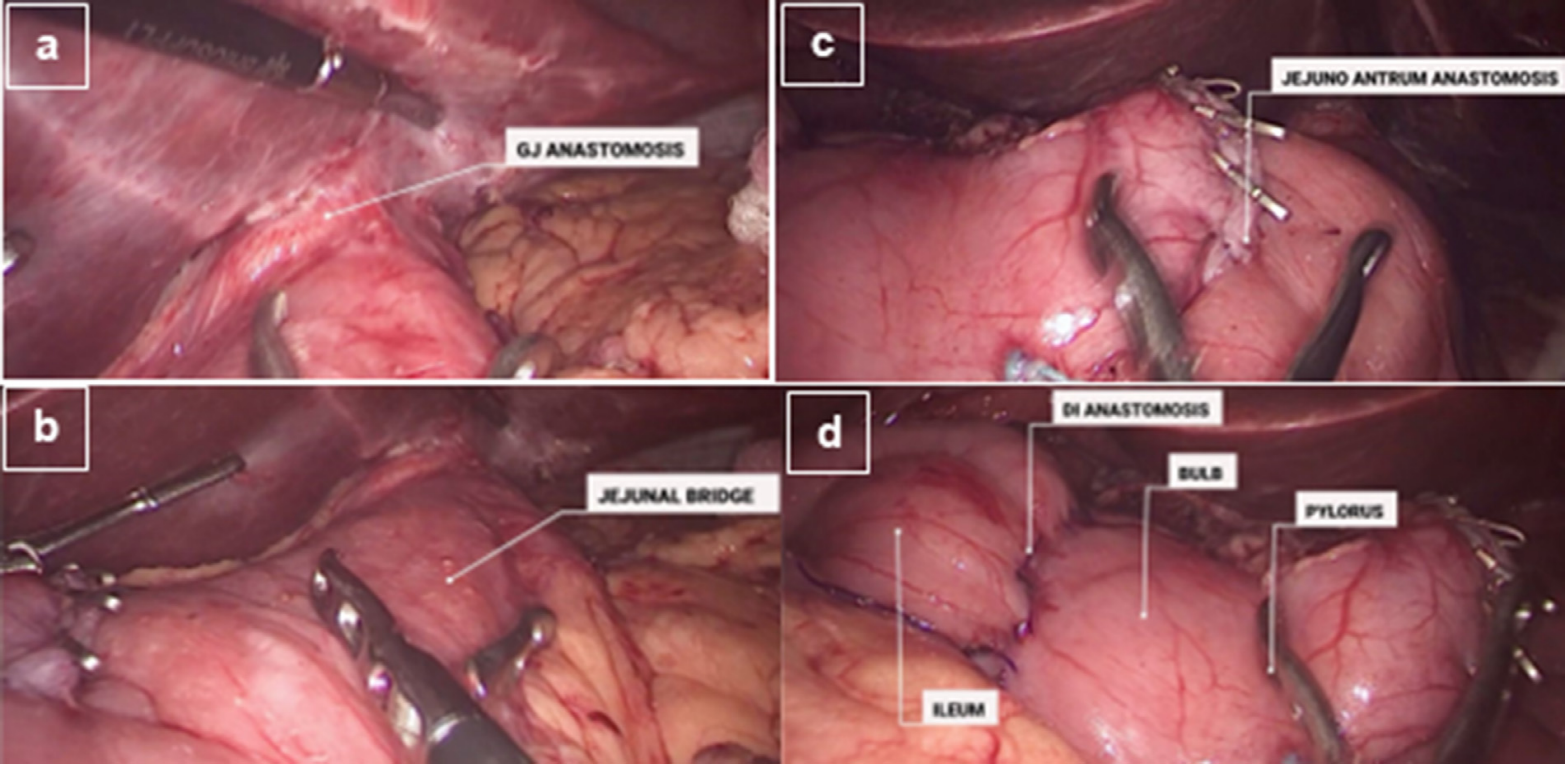
Final Aspects of the laparoscopic conversion of RYGB to SADIs with gastrogastric jejunal bridge. *GJ* Gastro-Jejunal; *DI* (duodenum-ileal)

• Step 1: Identified the gastroentero anastomosis, the left lobe of the liver, and here the alimentary limb from the previous bypass;
• Step 2: Cecum is identified, and 300 cm of ileal limb is measured, proximally from the ileal cecal valve (ICV), being fixed to the greater omentum with knot and clip. The remaining limbs are counted;
• **Step 3: Alimentary limb is identified up to the level of gastro-jejunal anastomosis;**
• **Step 4: Alimentary limb is transected 8 cm distally to the gastro-jejunal anastomosis;**
**• Step 5: The remnant stomach is dissected until the level of the antrum and transected horizontally, 3 cm above the pylorus;**
**• Step 6: Mechanical anastomosis is performed between the proximal alimentary limb and the posterior antrum wall;**
**• Step 7: The staple hole is closed in a single layer suture with PDS 3-0;**
• Step 8: The antropyloric and proximal duodenum arterial arcade are preserved;
• Step 9: The duodenum is dissected and transected 3 cm distally to the pylorus;
• Step 10: The ileal limb attached to greater omentum is released and a 2 cm duodeno-ileal anastomosis is performed, in a continuing two layers 3-0 PDS suture;
• Step 11: The Petersen space is kept open;
• Step 12: The remaining alimentary limb from RYGB is resected;
• Step 13: The anastomosis integrity is tested with methylene blue;
• Step 14: The specimens (excluded stomach, alimentary limb) are removed inside an endobag, after enlargement of left hypochondrium incision;
• Step 15: The trocars are removed, the aponeurosis is closed (sites of 12mm trocars and the enlarged wound) and the skin is sutured; No drains were left;

The Figs. 1, Fig. 2, Fig. 3 show the main phases presented in video to perform the conversion of RYGB to SADI-S with Gastrogastric Jejunal Bridge.

The proposed surgical conversion was performed laparoscopically, in a single stage procedure, that lasted 185 min (with gallbladder and candy cane removing in this patient) and had no intraoperative or postoperative complications.

RYGB is a safe and effective surgical procedure in the bariatric field, but presents 20 to 30% of obesity recidivism, in the long term (2). So, it is essential that we discuss a safe and effective surgical solution for these failures, a scarce topic in the current literature. The decision to convert the RYGB to SADI-S, by videolaparoscopy, was made based in the literature data that shows its greater metabolic potency then RYGB and a very close weight loss results, compared to BPD-DS, with fewer nutritional complications and being technically simpler (6). The gastrojejunal (GJ) anastomosis of the RYGB was preserved, avoiding adhesions in this region, even in larger gastric pouches (7). The proximal jejunal alimentary limb from RYGB (8 cm distal to GJ anastomosis) was used as a bridge between the gastric pouch and the antrum, avoiding the gastrogastric anastomosis, which has a significant incidence of fistulas, ulcers and refractory strictures (3,7). The conversion of RYGB to BPD-DS with a hybrid technique has been described in the literature (8). The lower tension and better vascularization in the jejuno-antral anastomosis probably reduce the risk of anastomotic complications. Cholecystectomy in this patient was performed due to gallstones presence and the “candy cane” was resected, which could be a cause of epigastric pain (9). The remnant stomach was resected, keeping the distal antrum and pylorus as part of gastric reservoir (3,8). After ensuring the adequate intestinal limb extension, the remnant jejunal alimentary limb was resected, preventing the possibility of bacterial overgrowth (10). The reimplantation of this hypertrophied alimentary jejunal limb in the intestinal transit was avoided, considering it could compromise weight loss (11). It was decided to preserve the antral and duodenal arterial arcades, minimizing risks of ischemia in the duodenal-ileal anastomosis (12). This anastomosis was done in a segment of ileum 300cm from the ICV, a measurement considered a little less effective in terms of weight loss, but nutritionally safer, compared to DS, considering all this segment will function as a common channel (13). The Petersen space was not closed, taking into account that internal hernias in SADI-S are rare (13,14).

The laparoscopically interposition of the proximal alimentary limb, from RYGB, between the gastric pouch and the antrum, acting as a bridge between them, in a conversion from RYGB to SADI-S, was a safe and feasible maneuver, in this case, done in a single stage procedure. The resection of the remnant alimentary limb, from the previous RYGB, makes the procedure easier.

The use of Gastrogastric Jejunal Bridge can be a reasonable tactic to convert RYGB to SADI-S, in case of weight regain. Furthermore, this maneuver could facilitate the conversion of RYGB to other bariatric techniques in which the sleeve is one of the surgical steps (Sleeve plus) or even for the isolated Sleeve itself.

## CRediT authorship contribution statement

**Victor Ramos Mussa Dib:** Conceptualization, Methodology, Investigation, Resources, Writing – original draft, Visualization. **Carlos Augusto Scussel Madalosso:** Visualization, Writing – review & editing. **Gabriela Trentin Scortegagna:** Writing – review & editing. **Rui Ribeiro:** Visualization, Writing – review & editing.

## Declaration of Competing Interest

Please tick the appropriate statement below (please donotdelete either statement) and declare any financial interests/personal relationships which may affect your work in the box below.

The authors declare that they have no known competing financial interests or personal relationships that could have appeared to influence the work reported in this paper.

The authors declare the following financial interests/personal relationships which may be considered as potential competing interests:

Please declare any financial interests/personal relationships which may be considered as potential competing interests here.

## Data Availability

No data was used for the research described in the article.

## Appendix Supplementary materials

Image, video 1

Image, video 2

Image, image 3

Image, image 4

## References

[1] Ozsoy Z, Demir E. (2018). Which Bariatric Procedure Is the Most Popular in the World?. A Bibliometric Comparison. Obes Surg [Internet].

[2] Obeid NR, Malick W, Concors SJ, Fielding GA, Kurian MS, Ren-Fielding CJ. (2016 Jan 1). Long-term outcomes after Roux-en-Y gastric bypass: 10- to 13-year data. Surgery for Obesity and Related Diseases.

[3] Keshishian A, Zahriya K, Hartoonian T, Ayagian C. (2004 Oct). Duodenal switch is a safe operation for patients who have failed other bariatric operations. Obes Surg.

[4] Moon RC, Alkhairi L, Wier AJ, Teixeira AF, Jawad MA. (2020 Oct). Conversions of Roux-en-Y gastric bypass to duodenal switch (SADI-S and BPD-DS) for weight regain. Surg Endosc.

[5] Dib V, Madaosso C, Scortegagna G, Ribeiro R. (2022 Aug). Conversion from Roux-En-Y Gastric Bypass to Sadi-S, with a Gastro-Gastric Jejunal Bridge as a Treatment of Obesity Recidivism: Case Report. International Journal of Science and Research (IJSR) [Internet].

[6] Surve A, Zaveri H, Cottam D, Belnap L, Medlin W, Cottam A. (2016 Nov). Mid-term outcomes of gastric bypass weight loss failure to duodenal switch. Surg Obes Relat Dis.

[7] Parikh M, Pomp A, Gagner M. (2007). Laparoscopic conversion of failed gastric bypass to duodenal switch: technical considerations and preliminary outcomes. Surg Obes Relat Dis.

[8] Topart P, Becouarn G. (2016 Nov 1). One-stage conversion of Roux-en-Y gastric bypass to a modified biliopancreatic diversion with duodenal switch using a hybrid sleeve concept. Surgery for Obesity and Related Diseases [Internet].

[9] Khan K, Rodriguez R, Saeed S, Persaud A, Ahmed L. (2018 Oct 1). A Case series of candy cane limb syndrome after laparoscopic Roux-en-Y gastric bypass. J Surg Case Rep [Internet].

[10] Miao D. (2017). Small Intestinal Bacterial Overgrowth in Modern Bariatric Surgery. Bariatric Times.

[11] le Roux CW, Borg C, Wallis K, Vincent RP, Bueter M, Goodlad R (2010 Jul). Gut hypertrophy after gastric bypass is associated with increased glucagon-like peptide 2 and intestinal crypt cell proliferation. Ann Surg.

[12] Mercado M, Cheng Q, Liu D, Loi K. (2022).

[13] Admella V, Osorio J, Sorribas M, Sobrino L, Casajoana A, Pujol-Gebellí J. (2021). Direct and two-step single anastomosis duodenal switch (SADI-S): Unicentric comparative analysis of 232 cases. Cir Esp.

[14] Surve A, Cottam D, Horsley B. (2020 May). Internal Hernia Following Primary Laparoscopic SADI-S: the First Reported Case. Obes Surg.

