# Supplementary figures and images for: Conversion of Roux-en-Y gastric bypass to single anastomosis duodenal ileal bypass with sleeve gastrectomy with gastrogastric jejunal bridge
Source: MethodsX. 2022 Dec 16;10:101971. doi: 10.1016/j.mex.2022.101971 (PMC9808024; doi:10.1016/j.mex.2022.101971)

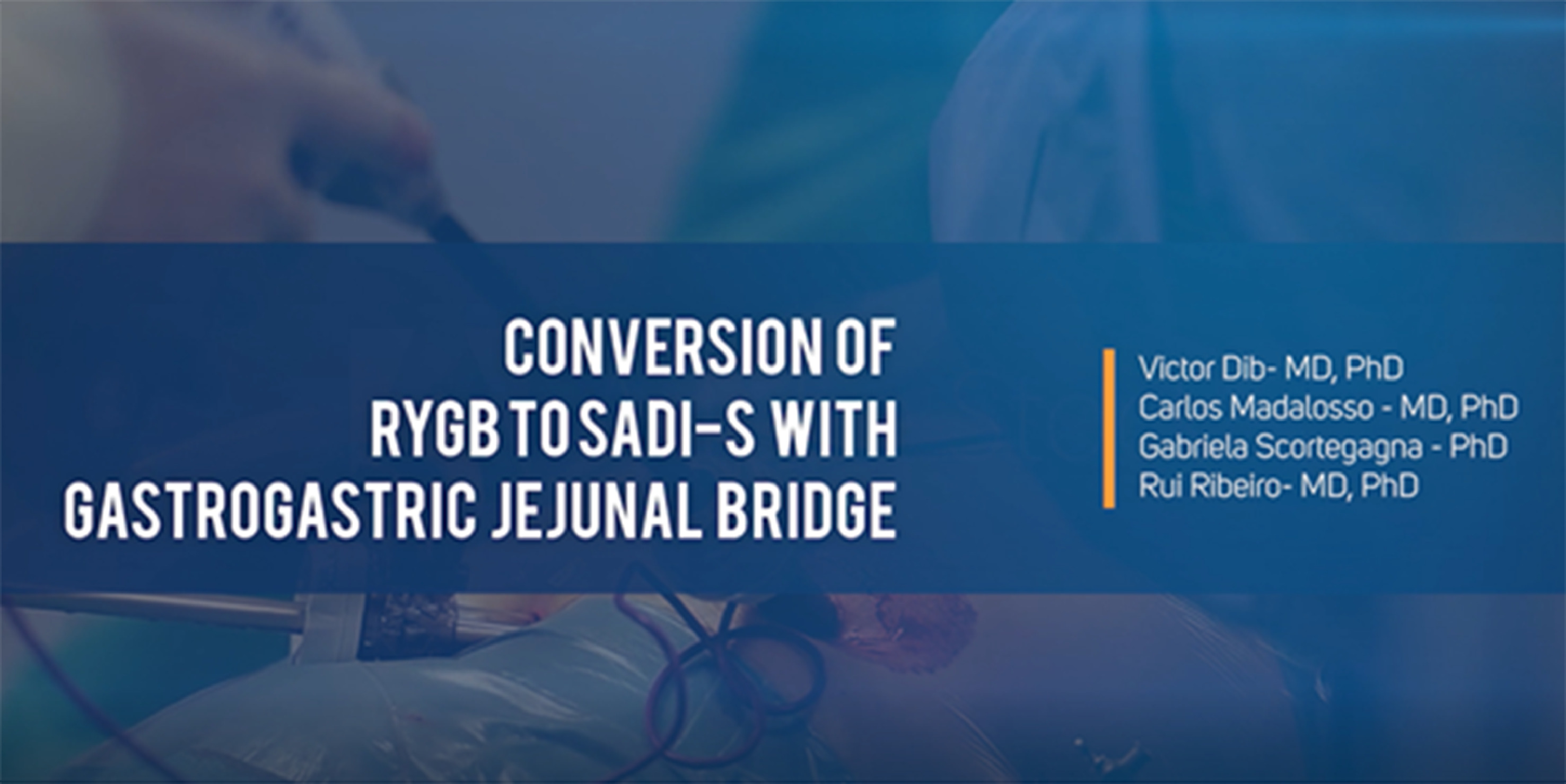

Supplement: Supplementary file 3 [file mmc3.jpg]

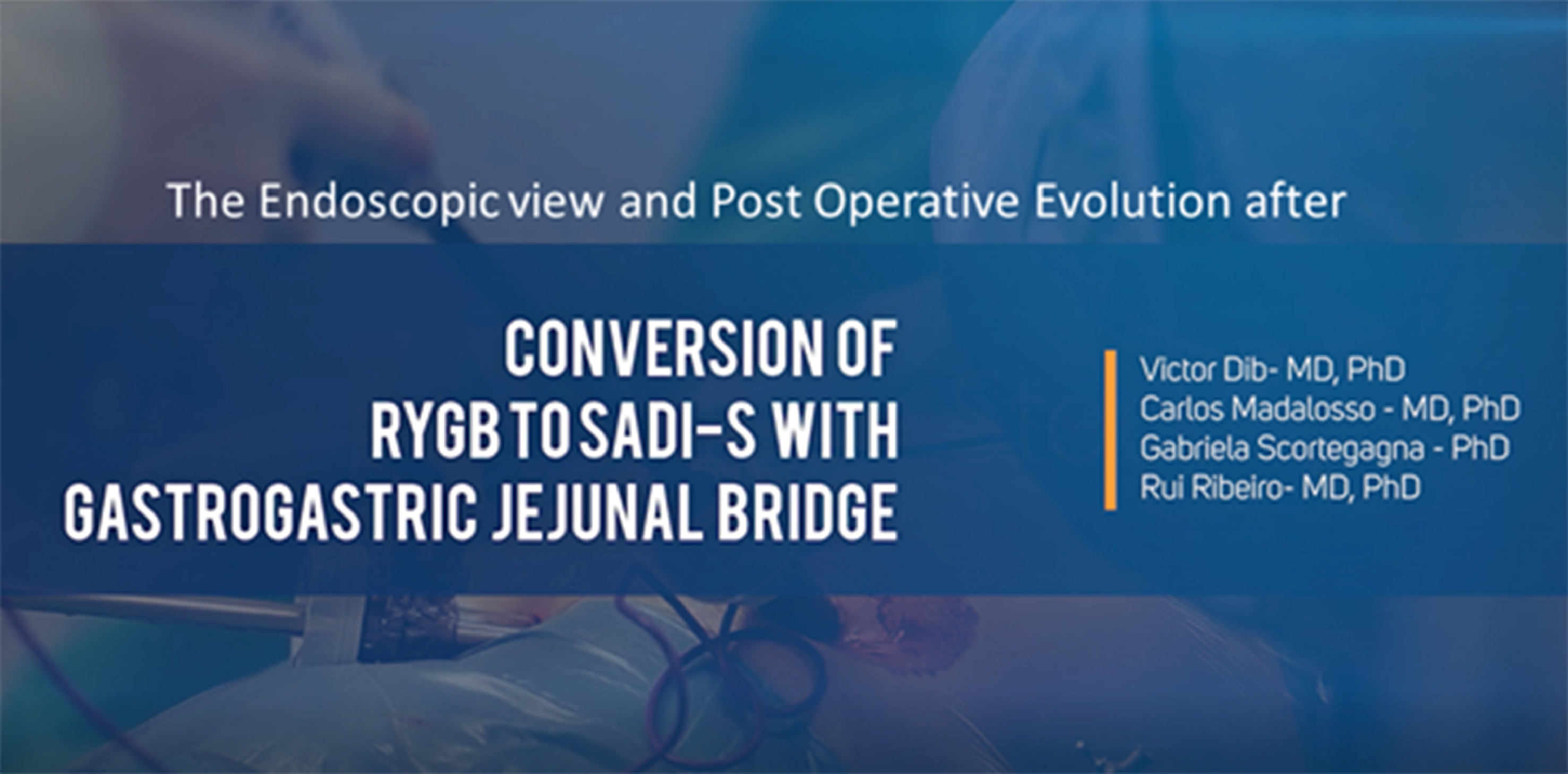

Supplement: Supplementary file 4 [file mmc4.jpg]
